# Gonococcus Infection in Infants

**Published:** 1903-12-05

**Authors:** 


### GONOCOCCUS INFECTION IN INFANTS.

In the light of fuller experience, it has become manifest that the gonococcus has not been given the importance it deserves as a pathogenic organism. It has been shown that this parasite is capable of growing in almost any tissue of the body, although the urogenital tract appears to offer the most suitable nidus. It has also been demonstrated that the gonococcus can give rise to epidemic illness. In drawing attention to the latter point, Dr. R. B. Kimball[1] states that vulvo-vaginitis is the bane of all institutions (in New York) in which infants and children are grouped together. The infection when once introduced spreads with great rapidity, attacking practically all the females, and unless energetic precautions are taken, some of the males. During last year there were 600 admissions to the public wards in the Babies' Hospital, New York, and among these patients there were 70 cases of vulvo-vaginitis and 10 cases of arthritis, due to the gonococcus. A large number of these were infected after becoming inmates of the hospital. As a result of this experience, it has been made a routine practice to take a smear from the vaginal secretions of every girl admitted, and to examine it for the gonococcus before receiving the patient into the wards. On the least suspicion of a vaginal discharge occurring in a ward patient another microscopical examination is made from every child. In dealing with cases in the hospital it has been found that only complete isolation will certainly prevent the disease from spreading, and this is especially the case among infants, who require so much more handling than older children.

Dr. Kimball believes that there is a remarkable susceptibility in infant life to gonococcus infection, and he states that this predisposition is inversely proportional to the child's age, being greatest in the very young babies.

In the same paper he reports eight cases of gonococcus pyæmia, in which the primary seat of infection was not manifest. All were under the age of three months. Seven of them were males. In everyone there was a suppuration arthritis in one or more joints, and the gonococcus was obtained from the pus in each case. Although the precise channel of *infection* in these cases could not be demonstrated, Dr. Kimball, for reasons which he gives, believes that the micro-organism gained entrance through the mouth—in one of the cases there was severe stomatitis before death. He points out that *infection* might be easily conveyed by the hands of a nurse to a baby during the cleansing of its mouth.

[1] Medical Record, November 14.

